# Molecular and Functional Phenotypes of Human Bone Marrow-Derived Mesenchymal Stromal Cells Depend on Harvesting Techniques

**DOI:** 10.3390/ijms21124382

**Published:** 2020-06-19

**Authors:** Sebastian G. Walter, Thomas M. Randau, Cäcilia Hilgers, El-Mustapha Haddouti, Werner Masson, Sascha Gravius, Christof Burger, Dieter C. Wirtz, Frank A. Schildberg

**Affiliations:** 1 Clinic for Orthopedics and Trauma Surgery, University Hospital Bonn, 53127 Bonn, Germany; sebastianwalter01@gmail.com (S.G.W.); thomas.randau@ukbonn.de (T.M.R.); caecilia.hilgers@ukbonn.de (C.H.); El-Mustapha.Haddouti@ukbonn.de (E.-M.H.); werner.masson@ukbonn.de (W.M.); sascha.gravius@ukbonn.de (S.G.); christof.burger@ukbonn.de (C.B.); dieter.wirtz@ukbonn.de (D.C.W.); 2 Clinic for Cardiothoracic Surgery, University Hospital Cologne, 50937 Cologne, Germany; 3 Department of Orthopaedics and Trauma Surgery, University Medical Center Mannheim of University Heidelberg, 68167 Mannheim, Germany

**Keywords:** mesenchymal stromal cells, phenotype, characterization, differentiation, harvesting technique, osteoimmunology

## Abstract

Mesenchymal stromal cells (MSC) harvested in different tissues from the same donor exhibit different phenotypes. Each phenotype is not only characterized by a certain pattern of cell surface markers, but also different cellular functionalities. Only recently were different harvesting and processing techniques found to contribute to this phenomenon as well. This study was therefore set up to investigate proteomic and functional properties of human bone marrow-derived MSCs (hBM-MSC). These were taken from the same tissue and donor site but harvested either as aspirate or bone chip cultures. Both MSC populations were profiled for MSC markers defined by the International Society for Cellular Therapy (ISCT), MSC markers currently under discussion and markers of particular interest. While classic ISCT MSC markers did not show any significant difference between aspirate and outgrowth hBM-MSCs, our additional characterization panel revealed distinct patterns of differentially expressed markers. Furthermore, hBM-MSCs from aspirate cultures demonstrated a significantly higher osteogenic differentiation potential than outgrowth MSCs, which could be confirmed using a transcriptional approach. Our comparison of MSC phenotypes obtained by different harvesting techniques suggests the need of future standardized harvesting, processing and phenotyping procedures in order to gain better comparability in the MSC field.

## 1. Introduction

Bone marrow (BM)-derived mesenchymal stromal cells (MSC) are multipotent cells that possess a unique capacity for self-renewal. Although autologous MSCs retain the ability to differentiate into cartilaginous, osseous and adipose tissue, the most prevalent clinical applications have been anti-inflammatory therapy and promotion of wound healing [1,2]. As research keeps focusing on MSCs as a potential source for clinical therapies (e.g., tissue engineering), comparability of studies relies on exact characterization of MSCs used for cultivation and further processing.

In the past, research has shown that there are differences in molecular cell characteristics when applying diverse harvesting techniques or collecting MSCs from different donor sites. These findings clearly showed that MSCs are difficult to compare and that most likely a complex orchestra of factors, starting with donor site, including the harvesting methodology used and ending with the way how cells were treated during cell culture, might have a dramatic impact on MSC phenotype. However, the MSC field is just beginning to understand how these methodological differences affect MSC biology. Even though a small selection of gene expression or proteome datasets was published in the last few years [3,4], there is still a great need for more systematic studies to tackle this problem. In this context, the MSC community also needed to agree that the classical MSC surface markers, such as CD73, CD90 and CD105, are insufficient for MSC characterization. Rather, the analysis of a broad, proteomic-like screening for surface markers, transcriptome clusters as well as description of functional properties, such as immunomodulatory capacity, regenerative potential, etc., is crucial for definitive characterization [5].

Today, the methods to harvest and purify MSCs are still very heterogeneous, differing between labs and researchers. This is highly critical and despite the potential cell biological consequences of such heterogeneity, this aspect does not get enough attention. There are several methods of harvesting MSCs: while adipose or umbilical cord-derived MSCs [6] are easy to obtain in practice [7], bone marrow aspiration and bone reaming remain the methods most often described as standard. While other authors have started to compare cells from different bones (femur versus iliac bones) with different anatomical and embryological properties, in this study, we derived MSCs from the same anatomical bone structure but used different harvesting techniques. Specifically, we compared bone marrow aspirate with bone chips from the femur. Both materials can be obtained very well during orthopedic and trauma surgery procedures and are therefore a reliable source for the production of a clinically relevant MSC product. Although in most cases it is easier to obtain bone marrow aspirates, significant amounts of bone fragments or bone chips are generated in some surgical procedures. Therefore, this direct comparison allowed us to evaluate whether these two very simple harvesting techniques have an impact on MSCs’ cellular phenotypes when brought into culture and how this would potentially affect clinical outcome.

Thus, the aim of this study was to investigate whether both different harvesting techniques from the same donor site result in the typical expression pattern of MSC markers and similar functional properties regarding osteo-, chondro- and adipo-genic differentiation behavior.

## 2. Results

### 2.1. Morphology and Proliferation Behavior of MSCs from Aspirate or Outgrowth Cultures

**Figure 1:**
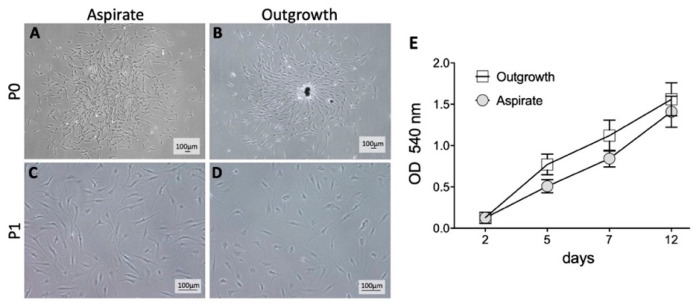
Morphology and proliferation behavior of mesenchymal stromal cells (MSC) from aspirate or outgrowth cultures. (**A**,**B**) The typical morphology of unpassaged MSCs is depicted. Adhered aspirate cells formed cell clones in contrast to an outgrowth culture with spare bone fragments as a source of cell growth. (**C**,**D**) All passaged cells appeared to be plastic adhered and spindle shaped. No relevant differences were observed. (**E**) Proliferation rate was measured by using an MTT Assay. Shown data were evaluated by optical density (OD) measurements. All isolated cells were viable and able to proliferate. No significant differences between both niches were detected.

BM-MSCs were obtained from the femoral bone during hip arthroplasty and harvested from outgrowth or aspirate cultures. Morphologically, there were no differences between outgrown and aspirated cells before and after passaging when analyzed by bright-field microscopy at P0 (Figure 1A,B) and P1 (Figure 1C,D). Also, no significant difference in optical density as pertains to cell growth was observed at any time points (Figure 1E), indicating that neither MSCs from aspirate nor MSCs from outgrowth cultures had any growth advantage.

**Figure 2:**
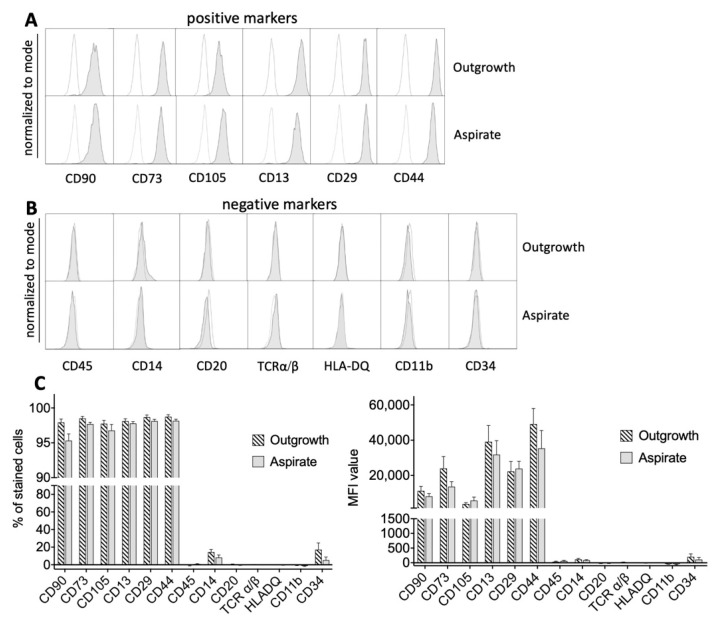
Expression of MSC surface markers defined by the International Society for Cellular Therapy (ISCT). (**A**) Both cells from aspirate and outgrowth cultures expressed ISCT MSC markers such as CD90, CD73, CD105, CD13, CD29 and CD44 without significant differences in expression levels (gray histograms). White histograms represent controls. (**B**) There was no significant difference for negatively expressed MSC markers defined by ISCT. (**C**) In correspondence with the previous findings, there were no significant differences between both groups regarding the percentage of stained cells for each marker or the corresponding mean fluorescence intensity (MFI) value, respectively. Only CD14 and CD34 showed a somewhat elevated expression signal.

To further analyze both MSC populations in more detail, cells were characterized using a variety of surface markers via flow cytometry. Interestingly, there was no difference in general MSC markers as defined by the International Society for Cellular Therapy (ISCT) [8]. There was no difference between MSCs from outgrowth and aspirate cultures in basic MSC marker expression of CD90, CD73, CD105, CD13, CD29 and CD44 (Figure 2A). Surface markers that were designated by definition as negative markers in MSCs, such as CD45, CD14, CD20, TCRα/β, HLA-DQ, CD11b and CD34, also showed no significant differences (Figure 2B). Further, to give a holistic impression of the MSC surface marker expression, the “% of stained cells” was analyzed to present the percentage of positive cells in the whole population and thereby indicate the relative number of cells that express a particular marker. In addition, we also analyzed the MFI (mean fluorescence intensity), which determines the relative amount of antigen that is present on the cell surface. Both parameters analyze the MSC population from a different perspective: a high MFI means that this cell population shows a high expression of the analyzed surface antigen. However, a high “% of stained cells” means that a lot of cells express this marker, but the overall expression could be low. That is why both parameters are very useful to give a thorough impression of distribution (how many cells express this marker) and expression level (how much is expressed) of a certain marker.

Interestingly, there were no differences in the percentage of stained cells and the mean fluorescence intensity (MFI) between MSCs from both groups (Figure 2C). For the positively expressed markers, more than 95% of the cells expressed the antigen of interest and exhibited a high MFI value, while for negatively expressed markers, no relevant signals were detected. Supplementary Figure S1 summarizes all analyzed surface markers as a heat map.

### 2.2. Controversially Discussed MSC Markers and Markers of Interest

**Figure 3:**
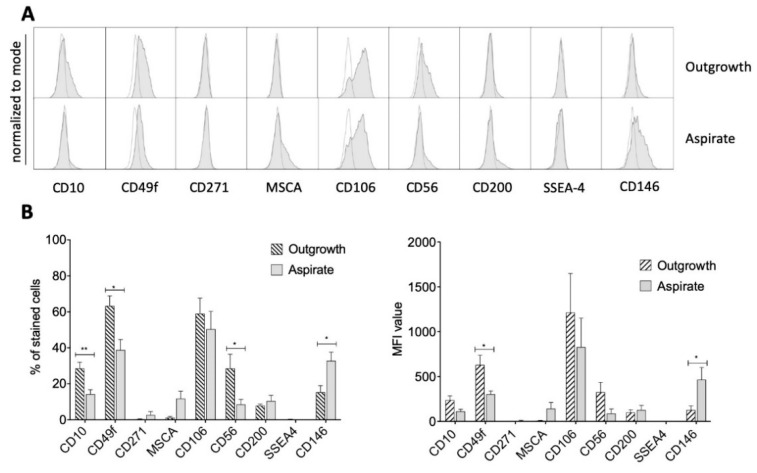
Expression of controversially discussed MSC markers. (**A**) Representative histograms (gray) indicated a distinct signal for CD49f and CD106. A weak signal was detected for CD10, MSCA, CD56, CD200 and both groups (aspirate and outgrowth) showed a lack of CD271 and SSEA4. White histograms represent controls. (**B**) Percentage of stained cells and their corresponding MFI confirmed the histograms. Furthermore, a statistically significant difference for the percentage of stained cells was detected for CD10, CD49f, CD56 and CD146, which was confirmed by significantly different MFI values for CD49f and CD146. * *p* < 0.05, ** *p* < 0.01.

In addition, several other potentially novel MSC markers were tested for differential expression profiles in both groups [9]. These markers are currently under discussion and are not yet ratified by the broad scientific community. MSCs from both groups showed no expression of CD271 and SSEA4 and only a weak signal for CD10, MSCA, CD56 and CD200 (Figure 3A,B).

Notably, both cell populations indicated distinct expression levels for CD49f and CD106 in histograms and there was a significant difference in the percentage of stained cells regarding CD10, CD49f, CD56 and CD146. While MSCs from outgrowth cultures expressed higher levels of CD10, CD49f and CD56, MSCs from aspirate cultures were associated with higher expression of CD146. Investigating the MFI level, significant differences were found for CD49f and CD146 (Figure 3B). While the former was significantly more expressed in outgrowth cells, CD146 showed an almost three-times increased expression in aspirate cells.

**Figure 4:**
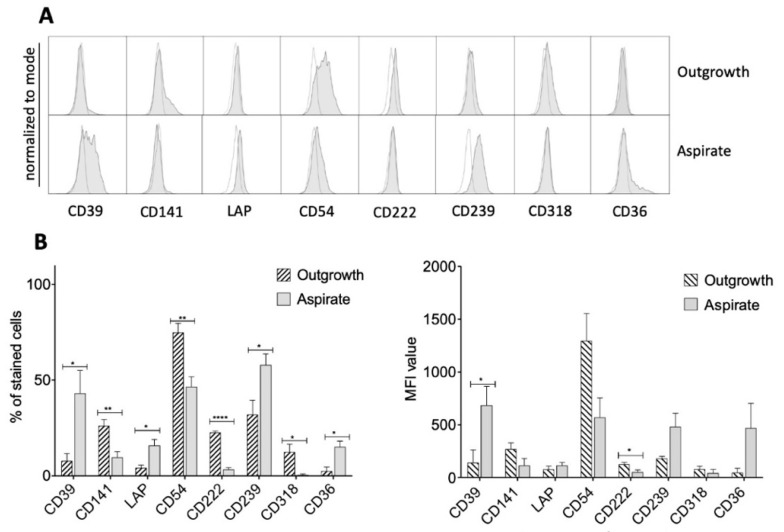
MSC markers of potential interest. (**A**) Representative histograms of further detected differences between outgrowth and aspirate cells (gray histograms). White histograms represent controls. (**B**) CD39, LAP, CD239, CD318 and CD36 showed low significance levels in percentages of cells stained for the given markers. For CD141 and CD54, this difference was medium, and for CD222, highly significant. MFI values indicate significant differences for CD39 and CD222 expression as well. * *p* < 0.05, ** *p* < 0.01, **** *p* < 0.0001.

In addition to the described surface marker panel, we had previously identified further markers in preliminary surface marker screenings, which are not common MSC markers but of potential interest regarding their biology. Therefore, these markers where further analyzed in this study in order to detect differences between aspirate and outgrowth cells [10,11,12,13,14,15,16,17]. Also using these surface markers, there were significant differences between the outgrowth and aspirate group (Figure 4). While CD39, LAP, CD239, CD318 and CD36 showed low significance levels, differential expression levels of CD141 and CD54 were medium but highly significant for CD222, as was shown by the percentage of stained cells and the MFI (Figure 4).

### 2.3. Multilineage Differentiation Capacities of Outgrowth and Aspirate MSCs

**Figure 5:**
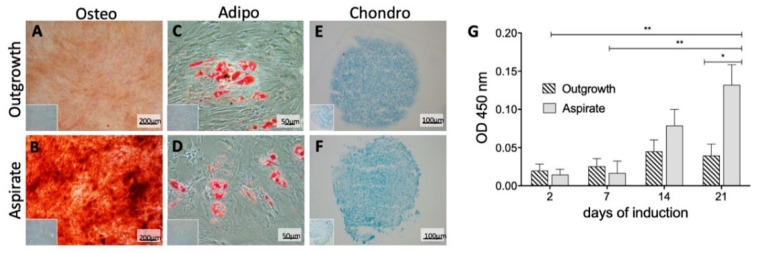
Differentiation capacity of MSCs from outgrowth and aspirate cultures. (**A**,**B**) Alizarin Red S, a staining for mineral deposits, indicates an osteogenic differentiation. Aspirate cells showed an increased signal compared to outgrowth cells. (**C**,**D**) Oil Red O is an indicator for lipids and visualizes adipocytes in red. Both niches were able to differentiate without significant difference. (**E**,**F**) Cell pellets with cartilaginous differentiation that were cut into 12 μm cryosections. Samples were subsequently stained with Alcian Blue to detect acid mucoids. Controls are indicated in the bottom left corners and in Supplementary Figure S2. (**G**) For quantification of the increased osseous differentiation potential of the aspirate cultures, the OD was measured at 450 nm at the indicated time points of osteogenic induction. Increased OD correlated with enlarged mineral deposits as an indicator of osteogenic differentiation. The bar charts show delta results of unstimulated cells subtracted from induced cells. After 21 days of osteogenic induction, aspirate MSCs exhibited a significantly higher OD than outgrowth MSCs. * *p* < 0.05, ** *p* < 0.01.

Comparable chondro- and adipo-genic differentiation characteristics were found in histological analysis (Figure 5C–F). However, their level of differentiation was relatively low, which might be due to the utilized isolation procedures or the specific microenvironment of the harvested bone, which potentially tweak MSCs to slightly favor the osteogenic differentiation.

Although, MSCs from both aspirate and outgrowth cultures were harvested from the same donor material, a significantly different osteogenic differentiation potential was observed after 21 days of osteogenic induction (Figure 5A,B).

In aspirate cultures, a significantly higher amount of mineral deposits was detected in alizarin red staining and, correspondingly, a significantly higher optical density (OD) at 450 nm was measured compared to outgrowth MSC cultures. At day 21, aspirate MSCs showed an OD that was almost three times as high as the outgrowth group (Figure 5G), therefore confirming its superior osteogenic potential.

**Figure 6:**
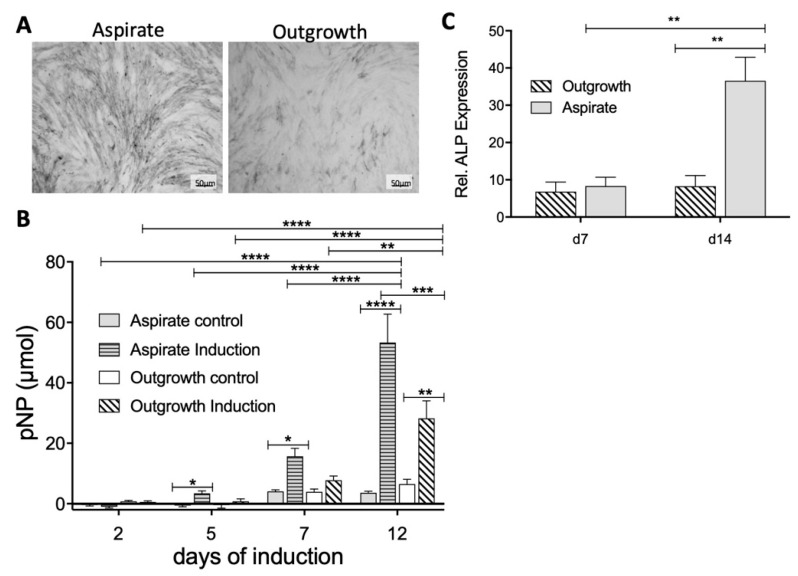
Comparison of alkaline phosphatase (ALP) expression by aspirate and outgrowth-derived MSCs. (**A**) ALP staining: The NBT/BCIP solution exhibited a black precipitate indicating the presence of ALP enzyme. MSCs from aspirate cultures showed a stronger ALP signal compared to outgrowth-derived MSCs. (**B**) Results of the ALP assay were evaluated by OD measurements. Starting at day 5, induced MSCs from aspirate showed higher ALP activity in comparison to the outgrowth-derived MSC group. (**C**) Relative gene expression level of ALP was determined using reverse transcriptase polymerase chain reaction (RT-PCR) for MSCs from both groups. * *p* < 0.05, ** *p* < 0.01, *** *p* < 0.001, **** *p* < 0.0001.

To further quantify the osteogenic differentiation, the alkaline phosphatase (ALP) activity was determined in both MSC populations by using histological staining as well as a fluorometric assay. Visualizing the ALP enzyme in an MSC monolayer culture showed a stronger ALP staining in MSCs from aspirate cultures compared to outgrowth cells (Figure 6A). Using a quantitative approach to measure ALP expression confirmed these findings by detecting significantly elevated ALP concentrations in MSCs from aspirate cultures in comparison to the corresponding controls (Figure 6B). This significant difference was detected first at day five of induction and increased multifold after seven days of osteogenic differentiation (Figure 6B). This trend was even stronger at day 12; however, by then, MSCs from outgrowth cultures also showed a significant increase. Of note, the outgrowth MSCs presented only half of the ALP activity in comparison to aspirate MSCs.

To even further validate the functional differences between MSCs from aspirate versus outgrowth cultures and to confirm the superiority of aspirate MSCs regarding their osteogenic potential, we performed RT-PCR analyses to quantify ALP mRNA expression (Figure 6C). Indeed, MSCs from aspirate culture showed significantly increased levels of ALP mRNA after 14 days of culture, which nicely confirmed our findings that MSCs from aspirate cultures not only show a distinct surface molecule repertoire but also an enhanced osteogenic differentiation potential.

## 3. Discussion

In current scientific debate, heterogeneity of MSCs is well acknowledged. One important aspect is whether MSCs from different tissues and MSCs exerting different phenotypes can be designated using the same term “MSCs” or if the general definition of MSCs needs to be revised [18,19].

Until now, major focus has been put onto different tissue sources. Yet, donor characteristics, harvesting methods and processing methods represent further crucial factors affecting differentiation potential of MSCs [20,21]. The influence of the latter, however, became the object of investigation only recently. In fact, all of the above-mentioned factors might impact MSC’s capacity to directed multilineage differentiation [22].

To date, most studies describing clinical and histological healing after MSC application did not characterize MSC phenotypes via a combined proteomic/flow cytometric and functional approach [23,24,25]. Flow cytometric cell surface proteomics represents a powerful tool to describe cell surface epitopes and allows the correlation of specific markers with functional features of the analyzed cells.

As shown in this study, isolation techniques have a major influence on MSC differentiation capacities and may be an important factor for success of translational studies; e.g., in the context of musculoskeletal tissue engineering, osteogenic differentiation potential is a crucial characteristic. MSCs that differentiate in osseous tissue may thus be the preferred cell source. It is, therefore, of high interest to define the optimal isolation methodology to generate the desired MSC phenotype. Obviously, there are several approaches to investigate the underlying MSC phenotype; however, the most common and meaningful are the characterization of surface marker expression as well as MSC differentiation potential.

The current study primarily aimed to analyze differences between MSCs generated from aspirate or outgrowth cultures. In this study, BM-MSCs that were aspirated demonstrated a better osseous differentiation capacity than BM-MSCs that were generated by outgrowth cultures. This showed that bone marrow aspiration is an important translationally relevant harvesting technique, which is further supported by the fact that this technique can more easily generate a decent amount of biomaterial and subsequently more MSCs in comparison to harvesting bone chips, which is clinically more limited in most cases.

Moreover, we found that MSCs isolated out of aspirate or outgrowth cultures showed both similarities and differences in terms of their surface marker expression. While some of these markers have been known to play a role in bone and MSC biology, for others, this association has not been so clear so far. For instance, it was shown that CD146 expression defines a subpopulation of human MSCs capable of bone formation and it was suggested to be suitable for clinical protocols of bone tissue regeneration. CD146^+^ MSCs were also shown to pursue trans-endothelial migration and homing to injured bone sites [26]. Migratory capacity of CD146^+^ MSCs is based on the exhibition of an enriched vascular smooth muscle cell phenotype and a smaller size and cytoskeletal morphology compared to CD146^−^ MSCs [27,28]. Furthermore, Kevorkova et al. identified the reduced expression of CD36 as a key factor contributing to reduced deposition of osseous matrix, which is in line with the phenotype of our aspirate group [29].

It is now tempting to mechanistically tie the different surface marker expression with the functional readout of the osteogenic differentiation, but at this point, this comparison is only an association and does not prove a link between surface marker and cellular function. However, from our point of view, the similarities and differences between these two isolation methods are of interest to the community and a discussion about potential association between surface markers and osteogenic differentiation could stimulate further studies to precisely analyze potential connections between surface marker expression and MSC function.

In this study, we investigated bone marrow-derived MSCs from the same tissue and donor site but harvested either as aspirate or bone chip cultures. This direct comparison is a novel aspect that has not been investigated so far. The study is a valuable contribution to the field, as it demonstrates the distinct impact of harvesting and processing methods on MSC quality and, thus, the importance of standardized procedures for the use of MSCs in clinical therapies. This is analogous to a study by Donnenberg and colleagues, who claimed a standard protocol for harvesting and subsequent processing in order to gain more comparability between different studies. In fact, it was suggested to investigate whether CD44^+^ cell sorting prior to cell culture would result in more homogeneous populations as expression of this marker was strongly correlated to expression of MSC markers as defined by ISCT [20,30].

Heterogeneity of MSCs may explain a broad spectrum of success rates in clinical studies as certain subpopulations of MSCs may be more suitable for certain biological applications and superior performance in translational settings than others [31]. This is because MSCs’ biological activity comprises immunomodulatory, anti-inflammatory and pro-regenerative capacities. Thus, in order to use and investigate MSCs in more detail, a more specific phenotyping of MSCs will be necessary for future studies.

In summary, for further studies investigating MSC-mediated bone regeneration, bone marrow-derived MSCs isolated by aspiration represent the source of choice because of their superior clinical relevance. This study additionally shows that a consensual standard protocol (including donor site, donor characteristics such as age, comorbidities, body mass index (BMI) and isolation as well as processing technique) urgently needs to be developed for the isolation and application of MSCs in order to achieve a better reproducibility and comparability of the results reported by different studies.

## 4. Materials and Methods

### 4.1. Tissue Donors and Isolation of Bone Marrow MSCs

Recruitment of subjects to obtain human bone marrow samples was approved by the local ethics committee (University Hospital Bonn, project ID: 122/09, approval date: 12 October 2009) and was conducted in accordance with the approved guidelines as well as the declaration of Helsinki. All included patients (*n* = 5) in this study were undergoing total hip arthroplasty due to primary coxarthrosis and showed no signs of congenital bone diseases, acquired diseases of the hematopoietic bone marrow, tumors or infections.

Bone marrow-derived MSCs were harvested during the procedure of hip replacement. When sawing the femoral bone, cells were either harvested by bone marrow aspiration or bone fragments were collected for outgrowth cultures. Bone chips that had to be removed for surgical reasons to perform total hip arthroplasty were washed with phosphate-buffered saline (PBS) to remove remaining blood. As fragments were not contaminated with connective tissue during surgery, no further cleaning steps were necessary.

Aspirated cells were isolated by scratching and flushing the spongious part of the femoral head or thin bone slices with PBS. The cell suspension was transferred onto a 70 µm filter and a Ficoll gradient was used in cases where a disproportionate number of erythrocytes was observed. Thus, the blood/PBS suspension was transferred on top of a 20 mL Ficoll and centrifuged for 30 min, 800 g, without break. The interphase was isolated, washed and placed into a cell culture flask.

Osseous fragments and aspirated cells were cultured under standard conditions at 37 °C/5% CO_2_ in Dulbecco’s modified eagle’s medium (DMEM) low glucose, containing 10% fetal bovine serum (FBS), 1% penicillin/streptomycin and 1% L-glutamin. According to our standard protocol, medium was changed twice a week. Within 1–2 weeks of incubation, a distinct outgrowth from bone fragments or cell clones from the aspirate cells were detected. To individualize cell aggregates, cells were trypsinized for 5 min with 0.05% trypsin-ethylenediaminetetraacetic acid (EDTA). Dense cells were passaged and frozen at p1 with freezing medium containing 10% dimethyl sulfoxide (DMSO), 40% FBS and 50% DMEM until further experiments were performed. After thawing, MSCs were further expanded for two more passages (p3) and then used for all downstream assays. For flow cytometric analysis, MSCs were trypsinized, washed with PBS and filtered to generate a single cell suspension. For all other assays, MSCs were cultured as monolayer.

### 4.2. Phenotypic Analysis of MSCs

Phenotypic surface marker expression analysis of human MSCs was performed using flow cytometry as described previously [32]. Briefly, cells were resuspended in PBS with 1% FBS/2 mM EDTA and were stained with saturating concentrations (1:25 dilution) of antibodies (Miltenyi Biotec, Bergisch Gladbach, Germany) for 20 min. Doublets and dead cells were excluded from the analysis. Unstained cells and isotype antibodies were used as controls. Flow cytometry data were acquired on a MACSQuant Analyzer 10 flow cytometer (Miltenyi Biotec, Bergisch Gladbach, Germany) and analyzed using FlowJo v10 (BD Biosciences, Heidelberg, Germany). The following antibodies (Miltenyi Biotec, Bergisch Gladbach, Germany) and clones were used: CD90 (DG3), CD73 (AD2), CD105 (43A4E1), CD13 (REA263), CD29 (TS2/16), CD44 (DB105), CD45 (REA119), CD14 (TÜK4), CD20 (LT20), TCRα/β (BW242/412), HLA-DQ (REA303), CD11b (M1/70.15.11.5), CD34 (AC136), CD10 (97C5), CD49f (GoH3), CD271 (ME20.4-1.H4), MSCA (W8B2), CD106 (REA269), CD56 (AF12-7H3), CD200 (OX-104), SSEA-4 (REA101), CD146 (541-10B2), CD39 (MZ18-23C8), CD141 (AD5-14H12), LAP (CH6-17E5.1), CD54 (REA266), CD222 (REA187), CD239 (REA276), CD318 (REA194), CD36 (AC106).

### 4.3. Real-Time Polymerase Chain Reaction

Total RNA was extracted from both outgrowth and aspirate MSCs using TRIzol Reagent (Ambion, Life technologies, Darmstadt, Germany) at indicated time points. Cells were washed with PBS, lysed in TRIzol and chloroform/isopropanol (ratio 24:1) (PanReac AppliChem, Darmstadt, Germany) was added. After centrifugation, the upper phase containing RNA was collected and precipitated by adding isopropanol and washed twice in ethanol (80%). RNA (1 µg) was reverse transcribed using Transcriptor First Strand cDNA Synthesis Kit (Roche Diagnostics GmbH, Mannheim, Germany) and RT-PCR was performed using LightCycler 480 SYBR Green I Master according to the manufacturer’s instructions (Roche Diagnostics GmbH, Mannheim, German). Data analysis was performed using the delta-delta-Ct (ddCT) method by normalization to 18S rRNA and the corresponding control samples without differentiation. Previously published primers were used to analyze ALP expression [33].

### 4.4. Analysis of MSC Differentiation Potential

MSCs were differentiated into the osteo-, adipo- and chondro-genic lineages, as described previously [32]. For osteogenic differentiation, MSCs were induced through high-glucose DMEM medium supplemented with 0.1 µM dexamethasone, 10 mM β-glycerophosphate disodium salt hydrate, and 50 µM 2-phosphate-L-ascorbic acid trisodium salt (Sigma Aldrich, Darmstadt, Germany). Induction towards the adipogenic lineage differentiation was performed by supplementing culture medium with 1 µM dexamethasone, 1 µM insulin, and 200 µM indomethacin (Sigma Aldrich, Darmstadt, Germany). The chondrogenic differentiation was performed as cell pellet culture using high-glucose DMEM medium supplemented with 1 µg/mL insulin, 1 ng/mL transferrin, 1 ng/mL sodium selenite, 0.1 µM dexamethasone, 50 µM 2-phosphate-L-ascorbic acid trisodium salt and 10 ng/mL transforming growth factor beta-1 (TGF-β1) (Sigma Aldrich, Darmstadt, Germany). All differentiation assays were performed for 21 days and culture medium lacking supplementation was used as control. All differentiated samples were fixed with 4% paraformaldehyde (PFA) before further treatment. Chondrogenic cell pellets were cut into 12 µm cryosections (Microm 550, Thermo Scientific, Schwerte, Germany).

For histological analysis, cells were stained with Alizarin Red S (Sigma Aldrich, Darmstadt, Germany) for evaluation of osteogenic differentiation or Oil Red O staining (Sigma Aldrich, Darmstadt, Germany) in order to determine adipose differentiation, as described previously [32]. For determination of chondrogenic cell differentiation, MSCs were stained with Alcian Blue (Sigma Aldrich, Darmstadt, Germany), as described previously [32].

For further quantification of osseous cell differentiation, MSC samples at a density of 1 × 10^4^ cells/cm^2^ were treated with 5-bromo-4-chloro-3-indolyl phosphate (BCIP)/nitro blue tetrazolium (NBT) substrate system (Dako, Hamburg, Germany) according to the manufacturer’s instructions to quantify the presence of the ALP enzyme. Further, alkaline phosphatase (ALP) activity was determined with the help of 4-Methylumbelliferyl phosphate disodium salt (MUP) substrate using a fluorometric assay kit according to the manufacturer’s instructions (BioVision, Inc., CA, USA). The absorbance was measured at 360 nm using a microplate reader (TECAN, Magellan, Switzerland).

### 4.5. Statistical Analysis

Statistical tests were performed with Prism 7 (GraphPad, La Jolla, CA, USA) using a two-tailed, unpaired Student’s *t*-test with a 95% confidence interval or two-way analysis of variance (ANOVA) assuming Gaussian distribution. Significance levels are marked as * *p* < 0.05, ** *p* < 0.01, *** *p* < 0.001 and **** *p* < 0.0001.

## Supplementary Materials

Supplementary Materials can be found at https://www.mdpi.com/1422-0067/21/12/4382/s1.

## Abbreviations

ALP	Alkaline phosphatase
BCIP	5-bromo-4-chloro-3-indolyl phosphate
BM	Bone marrow
CD	Cluster of differentiation
cDNA	Complementary desoxy ribonucleic acid
DMEM	Dulbecco’s modified eagle’s medium
DMSO	Dimethyl sulfoxide
EDTA	Ethylenediaminetetraacetic acid
FBS	Fetal bovine serum
GAPDH	Glyceraldehyde-3-phosphate dehydrogenase
hBM-MSC	Human bone marrow-derived MSCs
ISCT	International Society for Cellular Therapy
LAP	Latency-associated peptide
MFI	Mean fluorescence intensity
mRNA	Messenger ribonucleic acid
MSC	Mesenchymal stromal cells
MSCA	Mesenchymal stem cell antigen
MUP	4-Methylumbelliferyl phosphate disodium salt
NBT	Nitro blue tetrazolium
OD	Optical density
PBS	Phosphate-buffered saline
PFA	Paraformaldehyde
RNA	Ribonucleic acid
RT-PCR	Reverse transcriptase polymerase chain reaction
