# Supplementary material for: Molecular and Functional Phenotypes of Human Bone Marrow-Derived Mesenchymal Stromal Cells Depend on Harvesting Techniques
Source: Int J Mol Sci. 2020 Jun 19;21(12):4382. doi: 10.3390/ijms21124382 (PMC7352273; doi:10.3390/ijms21124382)
Supplement: Supplementary file 1 [file ijms-21-04382-s001.pdf]

# Supplementary figures

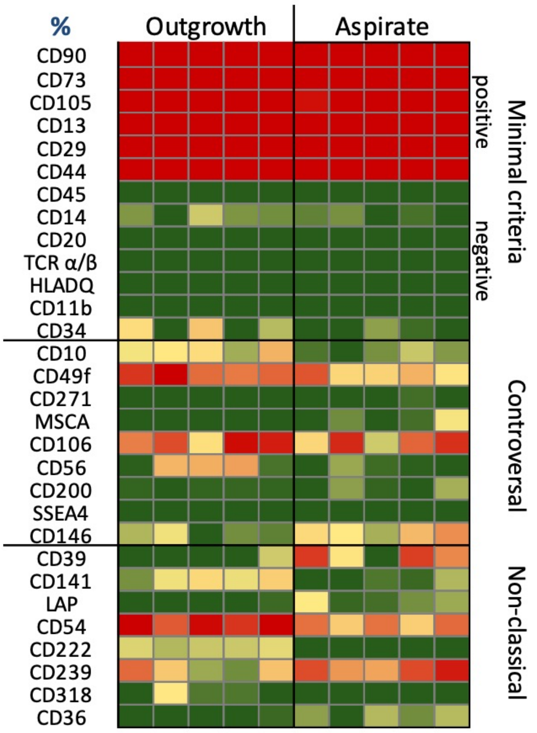


**Supplementary figure 1. Heat map of expression level of all discussed markers.** Depicted are the ISCT minimal criteria (positive and negative), controversially discussed markers, as well as non-classical MSC markers discussed in this study. Red color indicates high expression, green color low expression.

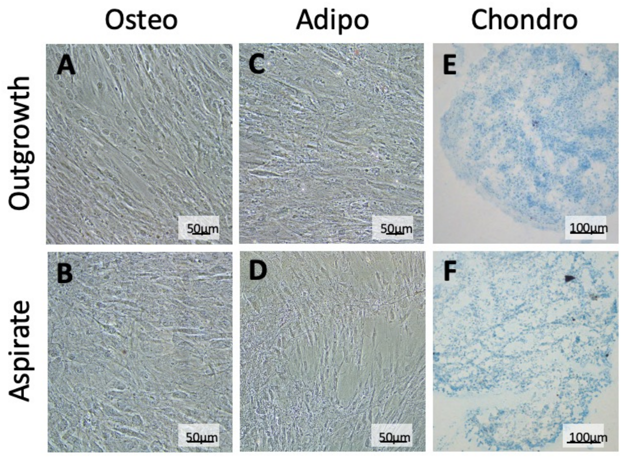


**Supplementary figure 2. Controls for MSC lineage differentiation from outgrowth and aspirate cultures.** MSCs were differentiated into the osteo-, adipo- and chondrogenic lineages. All differentiation assays were performed for 21 days and culture medium lacking supplementation was used as control. Resulting samples were fixed with 4% PFA before further treatment. Chondrogenic cell pellets were cut into 12 µm cryosections. **(A/B)** For evaluation of osteogenic differentiation, cells were stained with Alizarin Red S. **(C/D)** Adipose differentiation was determined using Oil Red O staining. **(E/F)** Alcian Blue staining was used for analyzing the chondrogenic cell differentiation.
